# Reciprocity in Medicine

**Published:** 1897-12-04

**Authors:** 


### RECIPROCITY IN MEDICINE.

In view of the difficulties which have arisen from the want of reciprocity between England and various foreign countries in regard to medical qualifications, it is interesting to observe that in the United States the same difficulty exists between one State and another. It appears that physicians from New York and other large cities are in the habit of visiting the health resorts of New England, and there combining business with pleasure. This causes much heart-burning among the resident medical men, who complain that these physicians have no right thus to take the bread out of their mouths, seeing that New England men who visit New York are denied the right of practising there. As is pointed out, however, by the *Medical Record*, the chief obstacle in the way of reciprocity is the inequality of the standards of education and examination in different States, which is a very different matter from the questions which have arisen between English and Continental physicians. Between us and the Italians it is a question of race, of language, of customs, and, to a large extent, of modes of treatment.

